# Publisher Correction: Towards machine learning aided real-time range imaging in proton therapy

**DOI:** 10.1038/s41598-022-08155-7

**Published:** 2022-03-08

**Authors:** Jorge Lerendegui-Marco, Javier Balibrea-Correa, Víctor Babiano-Suárez, Ion Ladarescu, César Domingo-Pardo

**Affiliations:** Instituto de Física Corpuscular, CSIC-University of Valencia, Valencia, Spain

Correction to: *Scientific Reports* 10.1038/s41598-022-06126-6, published online 17 February 2022

The original version of this Article contained errors.

In Figure 10, the left image, showing a picture of the actual “i-TED detector”, was omitted. Furthermore, in Figure 12 the left graph, which shows the time distribution (log scale) of the *γ*-ray and neutron events registered in i-TED, was omitted.

**Figure 10:**
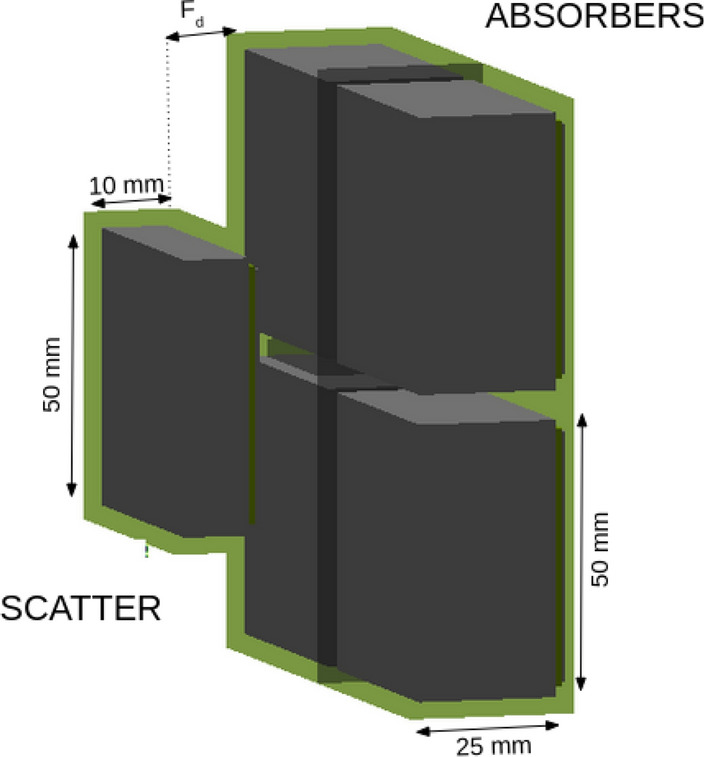
Left: i-TED detector consisting of one scatter and four absorber detectors in movable and parallel detection planes. Right: Schematic view of the same i-TED detector as implemented in GEANT4 indicating the dimensions of the LaCl33(Ce) crystals of the scatter and absorber planes.

**Figure 12:**
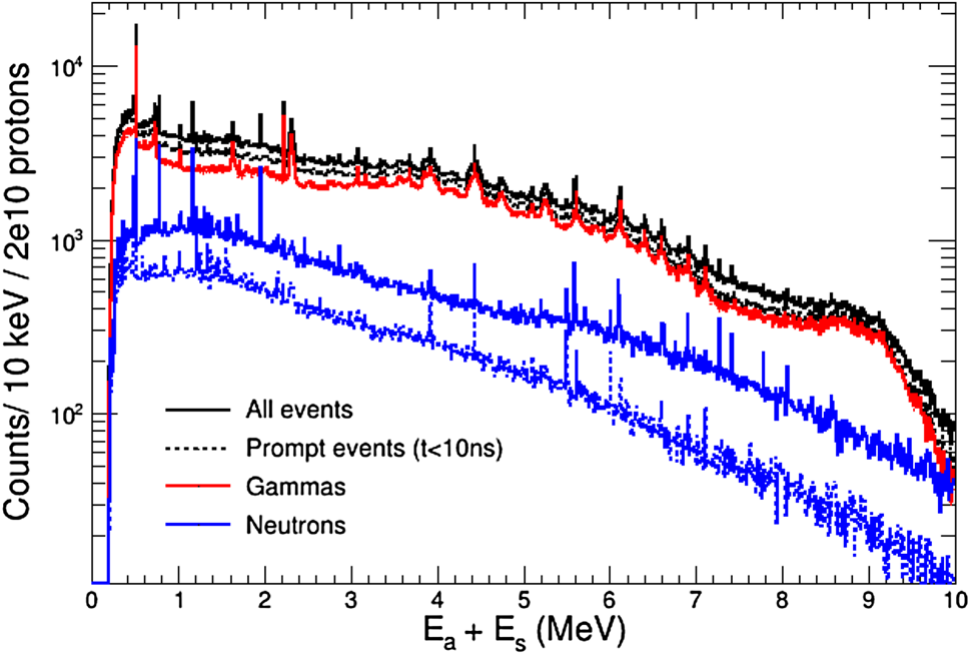
Left: time distribution (log scale) of the *γ*-ray and neutron events registered in i-TED, showing three distinct components which are separated by vertical dashed lines (see text for details). Right: add-back energy spectrum for all and prompt (t < 10 ns) events.

The original Figure 10 and Figure 12 and their accompanying legends appear below.

The original Article has been corrected.

